# Multiple disseminated pyogenic granuloma post–oil burning—Review literature

**DOI:** 10.1002/ccr3.3491

**Published:** 2020-11-06

**Authors:** Fariba Iraji, Minoo Jelvan, Zakiye Ganjei, Parvin Rajabi

**Affiliations:** ^1^ Department of Dermatology Medical School Isfahan University of Medical Sciences AL‐Zahra Hospital Isfahan Iran; ^2^ Department of Pathology Medical School Isfahan University of Medical Sciences AL‐Zahra Hospital Isfahan Iran

**Keywords:** Disseminated, lobular capillary hemangioma, oil burning, pyogenic granuloma

## Abstract

Disseminated pyogenic granuloma is a rare entity. Patients need reassurance for this benign condition and are advised about the risk of recurrence and the risk of scarring with a total surgical excision. Red‐flag diagnoses should be ruled out.

## Graphical abstract

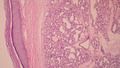

## 1 INTRODUCTION

Pyogenic granuloma (PG) is a common acquired vascular tumor and may appear mostly as a solitary lesions. Multiple disseminated PGs are a very rare form, and mostly are seen after traumas such as burn. We presented a new case with multiple PGs secondary to scald burn due to oil.

Pyogenic granuloma (PG), or lobular capillary hemangioma, is a common acquired proliferative vascular lesion of the skin and mucous membrane that may appear throughout childhood and adulthood. They occur most often on the face and distal extremities as a solitary, red nodule. PG has a pliable surface and bleeds easily ^1^, ^3^. While etiology of PG is unclear, trauma, infections, female sex hormones, viral oncogenes, microscopic arteriovenous anastomosing, and growth factors are considered as etiologic factors.^4^

Certain variants of PG have also shown an association with medications; reports suggest up to 30% of periungual PG are associated with medications but are also seen in association with other chronic dermatoses such as atopic dermatitis and psoriasis.^5^, ^6^

Disseminated pyogenic granulomas, although rare, have been documented to occur either spontaneously or after trauma such as burns. Certain medications are also implicated, including isotretinoin use in patients with severe nodulocystic acne and the use of granulocyte colony‐stimulating factor (G‐CSF) in immunodeficient patients.^7^, ^8^, ^9^

We presented a patient with multiple PGs developed after third‑degree scald burn due to oil, and this is the first report of disseminated PG post–oil burning. We also reviewed the literature and found 25 other cases that mostly caused by milk burning.^4^, ^23^

## 2 CASE REPORT

**Figure 1:**
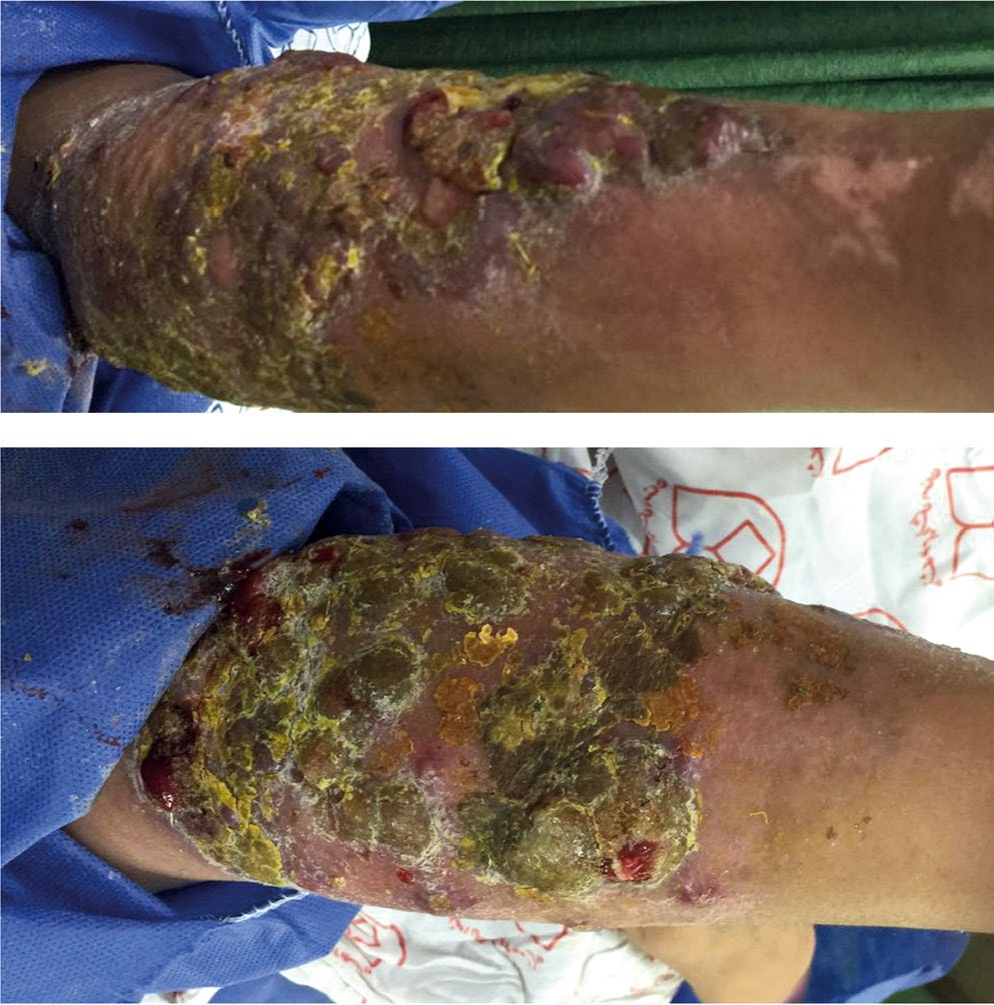
The appearance of multiple pyogenic granuloma on patient's forearm, 4 weeks after burn injury

A 30‑year‑old woman was referred to our department (Al‐Zahra Hospital; Referral Center for Treatment of Skin Diseases). The patient had 60% body surface third‑degree burn due to oil 4 weeks before. She was treated using daily dressing with silver sulfadiazine and intravenous antibiotic in a burn care center, and the burned skin in her thigh was successfully repaired with full‑thickness skin graft from the left forearm origin. During this period, 24 days after the burn injury, multiple papillomatoses and nodular lesions appeared periphery of the burn site and also around the donor site on her forearm (Figure 1). The lesions grew and bled easily.

**Figure 2:**
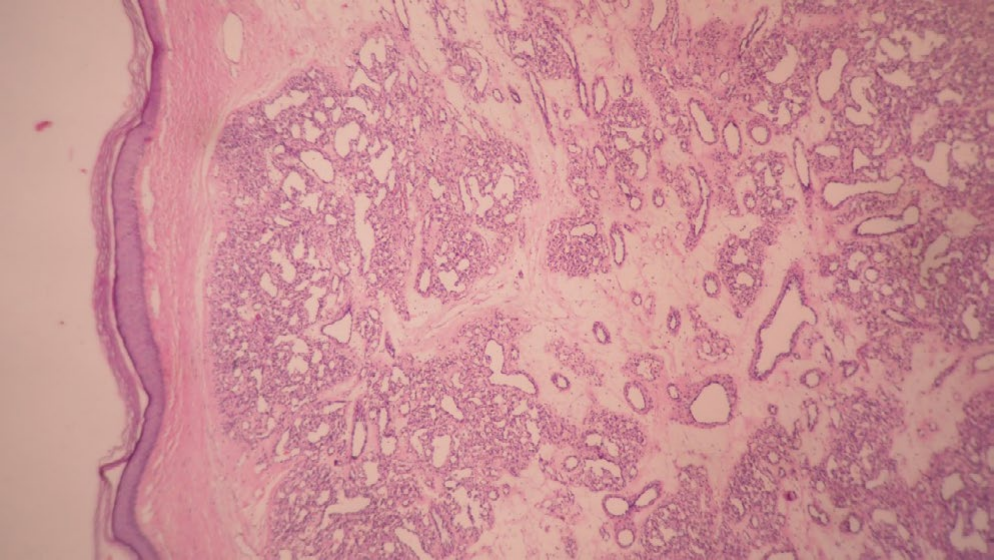
Histopathologic examination showed small capillaries with variable luminal diameters in an edematous stroma with scattered inflammatory cells, H&E (40x)

**Figure 3:**
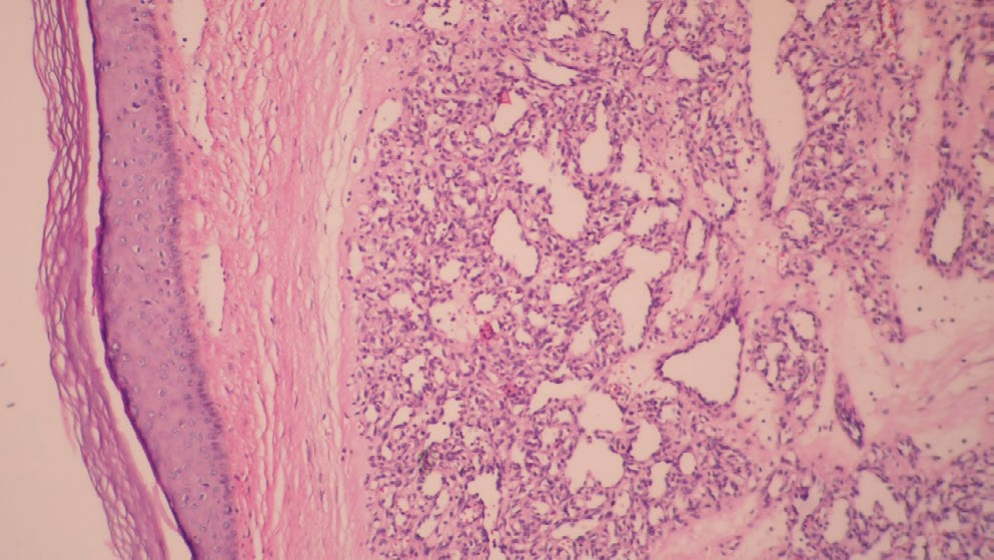
Histopathologic examination showed small capillaries with variable luminal diameters in an edematous stroma with scattered inflammatory cells, H&E (100x)

Laboratory investigation including complete blood count, liver, and renal function tests was within normal range.HIV and human T‑lymphotropic virus serology tests were negative. Blood and fresh tissue cultures for Bartonella spp. were negative. Histopathology examination showed hyperkeratosis, dermal edema, intense inflammatory cell infiltration(mostly lymphocytes and plasma cells), and bloody vessel proliferation (Figures 2 and 3).

The pathological features of this biopsy consisted with the clinical diagnosis of PG. Besides conservative treatment such as daily dressing and antibiotic, the excision of the lesions followed by electrosurgery of the base under local anesthesia was planned for her treatment and performed in the primary local facility. There was no evidence of recurrence 6 months later.

## 3 DISCUSSION

PG is a common acquired vascular tumor that is more common in the pediatric age group. The lesions present as rapidly growing papulonodules that are extremely friable, frequently ulcerate, and may bleed profusely with minor trauma. They appear mostly on the face, trunk, and distal extremities. While the etiology of PG remains unclear, the possible predisposing factors that affect the pathogenesis include trauma, infections, elevated female sex hormones level, viral oncogenesis, microscopic arteries venous anastomosis, growth factors, and drugs.^1^, ^2^, ^3^, ^4^, ^5^ Studies investigating specific angiogenic factors and signal transduction pathways have yet to implicate a single pathway for the pathogenesis of the lesion.

PG of different sizes occurs often as single lesions, and multiple disseminated lesions are rare form of PG, and in general, burns and widespread traumas may play a role in this form of PG. PG develops over the burned area within 1‐4 weeks following burns and may be infected with bacteria and fungi. As in other cases in the literature, there were 25 cases of disseminated PG following burn from 1978 to 2020.^4^, ^23^

The cases occurred approximately between 1 and 4 weeks following burning secondary to milk (nine cases), nine cases of scald burn, one case provoked by hot water, and four thermal burns or flames and two cases are not mentioned. Surprisingly, in our patient, the etiology was oil. In a majority of cases, the lesions developed following the second‑degree burn. As in our patient, conservative treatment or surgical excision was planned for them (Table 1).

**Table 1:** Reported pyogenic granuloma postburns

	Age/ sex	Causing agent	Degree of burn	Treatment
De kaminsky et al/ Argentina 1978 ^10^	15 months/F	Boiling milk	Second	Electrocoagulation
Momeni et al/ Iran/ 1995 ^11^	1‐5 years/M 5 years/F 35 years/F	Boiling milk	Second	Spontaneously resolved/Electrocoagulation in one case
Ceyhan et al/Turkey/ 1997 ^12^	18 months/ F	Boling Milk	Second	Surgical excision
Liao et al/ China/ 2006 ^13^	41 years/ M 19 years/M	Scald	Second	Conservative
Aliağaoğlu et al/ Turkey/ 2006 ^14^	5 years/F	Not mentioned	Second	Surgical excision
Bozkurt et al/ Turkey 2006 ^15^	2 years/ M	Boling milk	Second	Surgical excision
Diallo et al/ Senegal/2006 ^16^	8 months/ M 13 months/ M 13 years/M	Thermal burn	Second	Self‐healing
Ceyhan et al/ Turkey 2007 ^4^	17 months/M	Hot water	Second	Oral erythromycin
Ozbayoglu et al/ Turkey 2011 ^17^	8 years/M	flame	Second	Surgical excision
Shirol et al/India/2012 ^18^	42 years/F	Not mentioned	Second	Surgical excision
Durgun et al/Turkey/2013 ^19^	18 months/F 7 years/M	Hot milk	Second	Surgical excision
Zhao et al/China/2015/^20^	Five cases ranging from 15 months to 4 years	Scalding burn	second	Conservative/nonsurgical
Dastgheib et al/Iran/2016 ^21^	12 years/M	Boiling milk	second	Failed to follow‐up
Xu et al/China /2016^22^	4 years/F	Scalding burn	second	Conservative(Chinese herbal medicine)
Ashk Torab et al/2018^23^	15 months/F	Scalding burn	second	Conservative(herbal treatment)

Differential diagnosis includes amelanotic melanoma, squamous cell carcinoma, angiosarcoma, Kaposi sarcoma, hemangioma, bacillary angiomatosis, metastatic visceral malignancies, and granulation tissue..^19^The entities were ruled out both by clinical findings, histopathologic studies, and/or microbiological cultures. Conservative treatment including wound management and antibiotic could be chosen first, especially when large PG is on the face or other important areas of the body. As PG can involve the reticular dermis, pulse dye lasers, cauterization, and shave excision may not be able to reach the entire PG, and these methods of treatment have a recurrence rate of 43.5%.^24^ In our patient, the lesions were surgically excised followed by electrosurgery of the base, and no occurrence was observed during 6 months.

On a basic scale level, we think that the burn etiology and not the burn injury itself is important because all similar cases have the same etiology that may not be a coincidence, and milk proteins might cause the development of PG; Surprisingly, most reported cases due to milk are from Iran, Turkey and the habit of boiling raw milk at home instead of using pasteurized milk in urban areas may play a role.^21^

To the best of our knowledge, the most probable etiology is not a trauma or infection itself, but an idiosyncratic response to previous insults may play a role. Proposed mechanisms emphasize the importance of insults resulting in an imbalance of pro‐angiogenic and anti‐angiogenic factors, accompanying release of various proliferative and growth factors such as endothelial growth factor, fibroblast growth factor, and interleukin 1 B, which lead to a rapid proliferation of capillaries of a neovascular as PG.^15^, ^25^ Oil burning is reported in our case as a cause of disseminated PG for the first time, more research focusing on the etiology is needed, and the reasons why every trauma could not cause PG and why the same patient could not develop PG at later trauma are unclear.

## CONFLICT OF INTEREST

None declared.

## AUTHOR CONTRIBUTIONS

Fariba Iraji: Involved in study design and data collection. Minoo Jelvan: wrote the first draft of the manuscript. Parvin Rajabi: provided pathological photographs and interpreted pathological data. All authors: contributed to medical care, material preparation, and data collection; commented on previous versions of the manuscript.

## ETHICAL APPROVAL

Enrolled patients provided written informed consent.

## Data Availability

The data will be archived and will be available upon request after publication.
